# Improved topical delivery of curcumin by hydrogels formed by composite carriers integrated with cyclodextrin metal-organic frameworks and cyclodextrin nanosponges

**DOI:** 10.1016/j.ijpx.2024.100310

**Published:** 2024-12-03

**Authors:** Songting Li, Meng Long, Jiaqi Li, Yongtai Zhang, Nianping Feng, Zhicheng Zhang

**Affiliations:** a The People's Hospital of Yuhuan, 18 Changle Road, Yucheng Street, Yuhuan City, Zhejiang Province 317600, China; b Shanghai University of Traditional Chinese Medicine, 1200 Cailun Road, Pudong New Area, Shanghai 201203, China

**Keywords:** Hydrogel, Nanosponge, MOFs, Cross-linking, Transdermal drug delivery, Traditional Chinese medicine

## Abstract

Curcumin (CUR) is highly promising for topical therapeutic applications, but water-insolubility is one of the major challenges plaguing its drugability, while conventional lipid nanocarriers are limited by low drug-carrying capacity, many additives, and complex processes. In the current work, we constructed a composite carrier integrated with cyclodextrin metal-organic framework (γ-CD-MOF) and cyclodextrin nanosponge (β-CDNS), in which the γ-CD-MOF had 13.9 % drug loading and 267.1-fold increase in solubility in water for CUR, and the β-CDNS showed bioadhesion and further increasing drug solubility. The composite carrier (γ-CD-MOF@β-CDNS) significantly improved the in vitro release and transdermal permeation of CUR, and its limited water absorption properties and excellent bioadhesion create an advantage in the local administration of the drug for treating diseases with high exudate, which prevents the affected area from deteriorating the condition by counter-absorption of water from the formulation. Thus, this composite carrier contributes a new option to address the local delivery of insoluble drugs and offers a promising strategy for the clinical application of CUR.

## Highlights

• Increased water solubility of curcumin by 267.1-fold using γ-CD-MOF as a carrier.
• γ-CD-MOF@β-CDNS hydrogels are bioadhesive and increase the solubility of curcumin.
• γ-CD-MOF@β-CDNS significantly improved the transdermal permeation of curcumin.
• γ-CD-MOF@β-CDNS is suitable for topical administration in the presence of exudate.
• γ-CD-MOF@β-CDNS provides a new option for delivery of insoluble drugs.

## Graphical abstract

**Figure:**
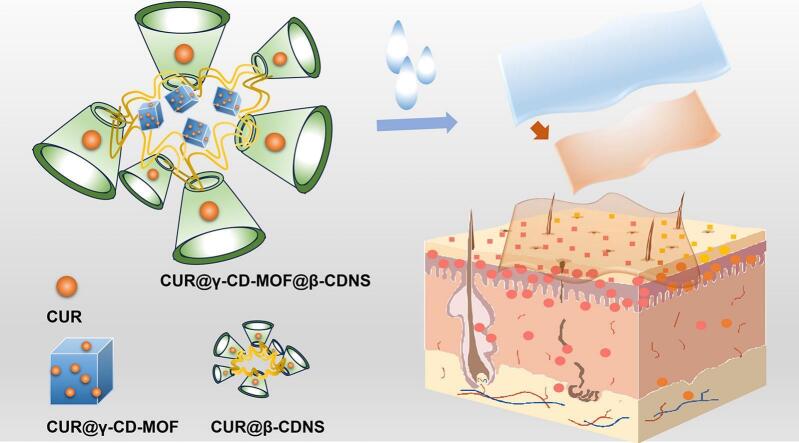

## 1 Introduction

Curcumin (CUR) is a polyphenolic compound with 1,7-diarylheptadiene as the basic skeleton (Prasain and Barnes, 2016), which has pharmacological effects such as antibacterial and anti-inflammatory, antioxidant, antitumor, hepatoprotective and choleretic, anti-Alzheimer's disease, and immunomodulation of the intestinal flora, and has no significant adverse effects (Nagargoje et al., 2024; Nunes et al., 2024). The application of curcumin for topical administration to relieve skin inflammation, treat psoriasis, and promote wound healing has been reported with promising efficacy (Kandaswamy et al., 2024; Poornima et al., 2024; Sghier et al., 2024). However, according to the Biopharmaceutics Classification System (BCS), CUR belongs to the BCS class IV with low solubility and low permeability, which is poorly water-soluble, rapidly metabolized in the gastrointestinal tract, poorly permeable to biomembranes, virtually non-absorbable when taken orally, and poorly permeable to percutaneous administration, which has hampered its conversion to clinical application (Lu et al., 2024; Sheng et al., 2023; Yusuf et al., 2024; Zhao et al., 2024).

Curcumin bioavailability can be improved by changing the dosage form or performing structural modification to increase its water solubility, but there are often various shortcomings: adding surfactants or organic solvents to the drug may cause potential toxicity; solid dispersions are poorly stabilized and are not suitable for long-term storage; and when the drug is made into a water-soluble salt, it is poorly targeted and prone to be in accumulating in tissues and organs (Ghosh and Sarkar, 2024). Traditional nano-preparations such as liposomes, micelles, microemulsions, etc. have the defects of small drug-carrying capacity, poor stability, and many additives (Liu et al., 2024a). Up to now, the development and application of curcumin-related products are very limited, therefore, seeking methods to effectively improve the water solubility and bioavailability of CUR is the key to promote promoting the development of the curcumin industry (Hajimirzaei et al., 2024; Wu et al., 2024).

Metal-organic frameworks (MOFs) are a class of crystalline porous materials with periodic framework structures formed by the self-assembly of organic ligands and metal ions (clusters) through ligand bonding (He et al., 2021). MOFs are characterized by large specific surface area, tunable structural ordering, easy functionalization, and biodegradability. Compared with traditional nano drug carriers, MOFs have the following advantages (Hefayathullah et al., 2024): (1) high porosity and specific surface area, and large drug-carrying capacity; (2) functional groups can be used to modify MOFs, or combined with the photomagnetic properties, to construct targeted molecules, to achieve targeted drug delivery and precise treatment; (3) the role of ligand bonding between the metal ions and organic ligands ensures that the biodegradability of the MOFs, and avoids toxic side effects caused by the accumulation of drug carriers; and (4) the molecular structure of MOFs is more variable, which can increase the solubility of the drug and improve the stability. When designing and selecting MOFs, the biocompatibility of the molecules should be emphasized to ensure that the organic ligands and metal ions composing the MOFs are free of toxic side effects on the human body. Zinc-based MOFs encapsulating CUR have been reported to enhance the antioxidant effect of drugs (Chen et al., 2024b; Liu et al., 2024b). Cyclodextrin (CD) and its derivatives are a class of commonly used carrier materials for drug inclusion. Such glucose ring-joined into a cylinder molecule with external hydrophilic and internal hydrophobic properties can encapsulate insoluble drugs and improve their solubility (Singh and Mahar, 2024). Among them, γ-cyclodextrin consists of 8 glucose units with large pores. γ-CD can act as an organic ligand to form a metal-organic framework with potassium ions, presenting a three-dimensional porous body-centered cubic structure (Qin et al., 2024). Potassium is a macronutrient in the human body, and the γ-CD-MOFs synthesized with γ-CD have excellent biocompatibility (Kru Kle-Be Rziṇa et al., 2024). γ-CD-MOFs possess a multiple pore-like structure with a large specific surface area and a sizable drug loading capacity. Therefore, in the current work, KOH and γ-CD were selected as raw materials to prepare MOFs (γ-CD-MOF) as carriers for CUR to solve the problem of its insolubility.

However, since γ-CD-MOF is a powdered solid with poor bioadhesion, which is unfavorable for local drug delivery, we therefore fabricated cyclodextrin nanosponges (CDNS) co-mingled with γ-CD-MOF as a composite carrier for CUR, which increases drug loading while adhering to skin or other tissues with the help of CDNS absorbing water to form a hydrogel and subsequently releasing the drug for local therapeutic effects. CDNS is a three-dimensional mesh nanocarrier based on the ultra-high cross-linking of α, β, and γ-cyclodextrin raw materials, which possesses various characteristics such as loose porosity, wide drug-carrying range, adjustable function, and stability (Pyrak et al., 2024). It can not only increase the solubility of insoluble drugs, increase the bioavailability of drugs, and regulate drug release, but also improve the stability of the encapsulated drugs, which has a broad application prospect (Garg et al., 2024). Cyclodextrin nanosponges (CDNS) have been applied to load a variety of drugs, such as improving the solubility of quercetin (Wu et al., 2021), increasing the chemical stability of camptothecin by decreasing the hydrolysis of lactones (Gigliotti et al., 2017), and improving the skin and mucosal permeability of resveratrol (Ansari et al., 2011), which can play an important role in improving the clinical application of drugs.

In this work, the high drug loading and excellent effect of potassium-based γ-CD-MOF on CUR to increase solubility in water were confirmed, and the hydrogel with weak mechanical properties of β-CD cross-linking-formed CDNS (β-CDNS) prepared by using epichlorohydrin as a cross-linking agent were characterized. The effect of the γ-CD-MOF and β-CDNS-formed hydrogel together as the carrier of CUR was evaluated in enhancing the transdermal penetration and local absorption of the drug, thus providing a case support for this novel composite carrier to improve the topical administration of insoluble drugs.

## 2 Materials and Methods

### 2.1 Materials

Epichlorohydrin (EPI) was purchased from Tokyo Chemical Industry (Tokyo, Japan), curcumin (CUR) and PBS were purchased from Dalian Meilun Biotechnology Co. Ltd. (Dalian, China). EDTA was purchased from Sigma Alarich (USA). Optimal cutting temperature compound (OCT) was purchased from Sakura Finetek Japan Co., Ltd. (Japan). The materials used for the preparation of MOFs included γ-CD, potassium hydroxide (KOH), methanol, polyethylene glycol (PEG) 20,000, isopropanol, methylene chloride, and other chemicals used in the current work were purchased from Sinopharm Chemical Reagent Company (China).

### 2.2 Experimental animals

Male healthy Kunming mice, SPF grade, weighing 18–20 g, were purchased from the Animal Experiment Center of Shanghai University of Traditional Chinese Medicine. The ethical approval number of experimental animals was PZSHUTCM2311080004, and the use license number was SYXK (Shanghai) 2020–0009. The temperature of the breeding environment was 18–25 °C, and the relative humidity was 40 %–70 %.

### 2.3 Detection of CUR

CUR was detected by LC-2010 A HPLC (Shimadzu, Japan) connected to an ODS C18 column (5 μm, 250 mm × 4.6 mm). The column was eluted with 0.5 % acetate aqueous solution: acetonitrile (40:60) at a flow of 1 mL/min at 30 °C, and the detection wavelength was 428 nm. The CUR concentration (x) in the range of 1–200 μg/mL showed a good linear relationship with the chromatographic peak area (y), and the regression equation was y = 77,822×-250,295, with the correlation coefficient *r* = 0.9998.

### 2.4 Preparation of γ-CD-MOF and CUR-loaded γ-CD-MOF (CUR@γ-CD-MOF)

1.62 g of γ-CD and 0.56 g of potassium hydroxide were dissolved in 50 mL of pure water, and 30 mL of methanol was added to form a white suspension, which was heated in a water bath at 50 °C until clarified. 650 mg of polyethylene glycol 20,000 was dissolved in 40 mL of methanol, mixed with the above clarified solution, and refrigerated at 4 °C for 24 h. The suspension was centrifuged at 3000 ×*g* for 5 min, and the precipitate was washed with isopropanol and dichloromethane sequentially, and dried under vacuum at 40 °C for 24 h, to obtain γ-CD-MOF. In addition, γ-CD-MOF was added to a curcumin 1,4-dioxane solution preheated to a set temperature at a concentration of 20 mg/mL and stirred for 2 h. The precipitate was then dried under vacuum at 45 °C for 24 h to obtain CUR@γ-CD-MOF.

To determine the drug loading, 10 mg of CUR@γ-CD-MOF was dissolved in 5 mL of methanol, all the drug was extracted by ultrasonication, and centrifuged at 7853 ×*g* for 5 min, and the mass of drug measured as a percentage of the mass of CUR@γ-CD-MOF was considered as the drug loading.

### 2.5 Preparation of β-cyclodextrin nanosponges (β-CDNS) and CUR-loaded β-CDNS (CUR@β-CDNS)

The preparation of β-CDNS was referred to the previous report, but the process parameters were optimized (Shende et al., 2015): 1.14 g of β-CD was dissolved in 4 mL of DMSO, 1 mL of triethanolamine was added as a catalyst, and 2.01 g of EPI was added under magnetic stirring at 600 rpm, and polymerized at room temperature for 30 min until a solid product was formed, and then extracted by refluxing with acetone for 48 h. The purified solid product was then dried under vacuum at 50 °C for 24 h. In order to evaluate the residual amounts of triethanolamine and DMSO, an Elementar Unicube instrument (Elementar Analysensysteme GmbH, Germany) was employed for detecting C, H, N, and S in β-CDNS. The temperatures of the combustion and reduction tubes were 1150 °C and 850 °C, respectively, and the helium and oxygen flow were 200 mL/min and 15 mL/min, respectively, both at a pressure of 1200 mbar. Detection was carried out at 65 °C with a thermal conductivity detector.

To prepare CUR@β-CDNS, CUR was dissolved in anhydrous ethanol or 1,4-dioxane, and β-CDNS was added with magnetic stirring at 50 °C and 600 rpm to the set duration, and then centrifuged at 7853 ×*g* for 5 min, and the precipitate was dried completely in vacuum at 50 °C. Another CUR@β-CDNS was dissolved in 1,4-dioxane, and centrifuged at 7853 ×*g* for 5 min, and the absorbance of CUR in the supernatant at 426 nm was measured by Spark 10 M microplate reader (TECAN, Switzerland), and the amount of drug load was calculated.

### 2.6 Preparation of β-CDNS containing CUR@MOF (CUR@γ-CD-MOF@β-CDNS)

200 mg of CUR was dissolved in 10 mL of 1,4-dioxane, 255 mg of γ-CD-MOF was added, 65 °C, 600 rpm magnetic stirring for 2 h, while 100 mg of β-CDNS was added, kept at 50 °C and stirred magnetically at 600 rpm for 10 h, and centrifuged for 5 min at 7853 ×*g*. The precipitate was vacuum dried at 50 °C to obtain CUR@γ-CD-MOF@β-CDNS.

### 2.7 Equilibrium solubility determination of CUR

An excess of the test sample was added to purified water, and this suspension system was placed in a shaker and processed at 37 °C for 12 h with a stirring speed of 150 rpm, and the supernatant was centrifuged at 7853 ×*g* for 5 min to determine the CUR.

### 2.8 Particle size determination

The test samples were dispersed in anhydrous ethanol, and the particle size was determined at 25 °C with a Nanozs 90 Malvern particle sizer (Malvern, UK).

### 2.9 Scanning electron microscopy (SEM) observation

The test samples were uniformly coated on the conductive adhesive, sprayed with gold treatment, and observed by Quanta 250 FEI scanning electron microscope (FEI, USA).

### 2.10 Powder X-ray diffraction (XRD) detection

The test samples were loaded on a slide of Smart Lab SE X-Ray Diffractometer (Rigaku, Japan) for CuKa target diffraction, with tube voltage and current of 40 kV and 40 mA, respectively, and scanned at an angle ranging from 3° to 40°, with a scanning step size of 0.02°, and scanning speed of 0.1 s per step.

### 2.11 Differential scanning calorimetry (DSC) analysis

The samples were analyzed using STA449F3 thermal analyzer (NETZSCH, Germany), heated from 25 °C to 400 °C at a ramping rate of 10 °C/min under nitrogen protection, and the DSC curves were recorded.

### 2.12 Fourier transform infrared spectroscopy (FTIR) determination

The test samples were pressed together with potassium bromide and the infrared absorption spectrograms were determined by scanning in the range of 4000–400 cm^−1^ using R330 Fourier transform infrared spectrometer (Thermo, USA).

### 2.13 Contact angle measurement

The test sample was pressed into a tablet and placed on the measuring table of the JY-82C Video Contact Angle Tester (Chengde Dingsheng Testing Machine and Inspection Equipment Co., Ltd., China), and 2 μL of ultrapure water was added by dropping with a microsyringe to measure the contact angle of the water droplet with the plane interface.

### 2.14 Moisture absorption test

The test samples of known weight were placed in weighing bottles, stored at 25 °C and 85 % relative humidity, and weighed at preset time points respectively. The moisture absorption rate was taken as the percentage of the weight gain of the moisture absorption to the original weight of the sample.

### 2.15 Examination of gel-forming property

100 mg of the test powder was placed in a 2 mL centrifuge tube, respectively, and 50, 80, 100, and 150 μL of pure water was added, and the gel was prepared by stirring evenly, and the centrifuge tube was inverted for 12 h to observe the adhesion and fluidity.

### 2.16 Rheological test

100 mg of the test powder was added to 50 μL of pure water to prepare a gel, and the rheological properties of the samples were determined by using an MCR101 rotational rheometer (ANTON PAAR, Austria) at 25 °C, with a shear rate in the range of 0.01–100 s^−1^, and the graphs of the changes in shear stress and shear viscosity with the increase in shear rate were recorded. The viscoelasticity of the samples was measured at 25 °C with the frequency ranging from 0.1 to 100 Hz, and the graphs of the changes of storage modulus and loss modulus with the increase of frequency were recorded.

### 2.17 Bioadhesion

Mice were anesthetized by intraperitoneal injection of 0.3 % pentobarbital sodium injection (0.2 mL/10 g), and the abdominal site was depilated by removing hair with an electric razor and humanely executed. The skin of the depilated area was peeled and the subcutaneous tissue was removed to obtain isolated skin. A gel was prepared by adding 100 mg of the test powder to 50 μL of pure water, and uniformly coated on one side of the stratum corneum of isolated mouse skin with an area of 1 cm^2^. The skin was fixed on a slide, and the slide was tilted at an angle of 45°, and the adhesion status of the gel on the skin was observed after 10 min, and then rinsed with purified water at a flow rate of 10 mL/min, and the amount of water used to completely rinse off the gel was recorded.

### 2.18 In vitro release test

The test sample containing 1 mg of CUR was placed into a dialysis bag (MWCO 8000–14,000 Da), and 40 mL of PBS (pH 7.4) containing 1 % sodium dodecyl sulfate and 0.5 % Tween 80 was used as the release medium (to meet the sink conditions), and shaken at 37 °C, 100 rpm in a shaker, and 1 mL of the sample was withdrawn at the pre-set time point, and supplemented with 1 mL of 37 °C fresh medium. The concentration of CUR in the resulting samples was determined and the cumulative drug release curve was plotted.

### 2.19 In vitro permeation test

The mouse skin stratum corneum was immobilized face up between the supply and receiving pools of the modified Franz diffusion cell. The test samples containing 2 mg of CUR (mixed in the ratio of 100 mg of test sample to 50 μL of purified water) were added into the supply cell separately. PBS containing 30 % PEG 400 was used as the receiving solution (to meet the sink conditions), and the transdermal permeation test was performed at 37 ± 0.5 °C and a magnetic stirring speed of 300 rpm. Samples of 1 mL were taken at preset time points and supplemented with the same volume of fresh receiving solution preheated to the same temperature. The CUR concentration of the collected samples was determined. The cumulative transdermal volume at each time point was calculated according to Eq. 1, and the time-cumulative transdermal amount of drug curve was plotted.

(1) $$\mathrm{Qn}=\frac{C_{n}V+\sum_{i=1}^{n-1}\times C_{i}\times V_{i}}{A}$$

where Cn and Ci are the drug concentration measured in the n-th and i-th transdermal sample respectively, V and Vi are the volume of receiving cell and sampling volume respectively, and A is the effective permeation area (cm^2^).

At the end of the transdermal permeation test, the residual drug on the skin surface was quickly rinsed off, fixed in formalin for 2 h, embedded in OCT, and quick-frozen in liquid nitrogen. The skin was cut longitudinally with a CM1950 frozen sectioning machine (LEICA, Germany), and the drug distribution in the skin was rapidly observed by Cytation5 Cell Imaging Multifunctional Detection System (Bio Tek, USA).

### 2.20 Statistical analysis

Data were analyzed using GraphPad Prism 9.3.0 software. The *t*-test was performed between two groups of data, and one-way ANOVA was used to analyze the differences among multiple groups, with *P* < 0.05 being a statistically significant difference.

## 3 Results and discussion

### 3.1 γ-CD-MOF as a carrier significantly increased the solubility of CUR in water

**Fig. 1:**
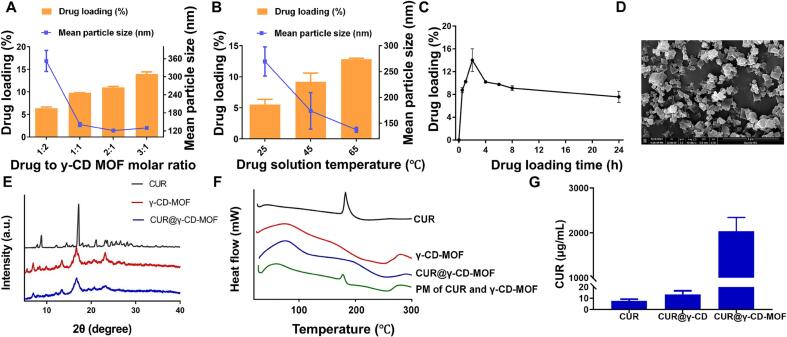
Preparation process optimization and characterization of CUR@γ-CD-MOFs. (A) Effect of drug to γ-CD-MOF molar ratio on particle size and drug loading of the carrier (*n* = 3). (B) Effect of drug solution temperature on particle size and drug loading of γ-CD-MOF (n = 3). (C) Effect of loading time on drug loading of γ-CD-MOF (n = 3). (D) SEM micrograph of CUR@γ-CD-MOFs. (E) X-ray diffraction patterns of CUR, CUR@γ-CD, and CUR@γ-CD-MOF. (F) The DSC curves of CUR, γ-CD-MOF, CUR@γ-CD-MOF, and physical mixture (PM) of CUR and γ-CD-MOF. (G) Equilibrium solubility CUR, CUR@γ-CD, and CUR@γ-CD-MOF in water (n = 3).

In conventional drug loading methods, drug concentration is an important factor affecting the amount of γ-CD-MOF loaded, and usually the drug concentration is positively correlated with the amount of drug loaded (Ruan et al., 2024a). In the current study, the drug loading was carried out in the range of 1:2–3:1 drug to carrier molar ratio, setting the temperature of the drug solution at 65 °C and the immersion time of the carrier in the drug solution at 2 h. The drug loading capacity of the resulting CUR@γ-CD-MOF gradually increased with the increase of the drug to carrier molar ratio, but its particle size decreased from 353 nm to about 130 nm (Fig. 1A). Due to the limited solubility of CUR in the solvents used, the molar ratio of drug to carrier was chosen to be 3:1, and the comparison of the effect of the drug solution temperature of 25–65 °C on the drug loading was continued. As the temperature increased, the drug loading and particle size of CUR@γ-CD-MOF showed an increasing and decreasing trend, respectively (Fig. 1B), while the drug loading was the highest when the drug solution was 65 °C, and meanwhile, the carrier particle size was the smallest, while the smaller particle size possessed a larger specific surface area, which is favorable for drug dissolution and in vivo absorption. Although the trend of the data suggests that higher temperatures may yield greater drug loading, it is accompanied by the challenge of rapid volatilization of organic solvents and reduced chemical stability of the drug at high temperatures (Forte et al., 2024). Further comparing the results of drug loading duration from 0.5 to 24 h, the drug loading of CUR@γ-CD-MOF first increased with time, reached a peak at 2 h, and then decreased, and then the decreasing trend slowed down after 8 h (Fig. 1C), suggesting that drug desorption and degradation of the drug from the carriers may occur with too much drug loading time. The above results are consistent with our previous report (Ruan et al., 2024b). The final preparation process was set as 3:1 drug to carrier molar ratio, the temperature of the drug solution was 65 °C, the loading time was 2 h, and the resultant product had a drug loading of 13.9 %, and a mean particle size of 130 nm.

The CUR@γ-CD-MOFs were in the shape of a cube with a smooth surface and a nanoscale distribution with a relatively homogeneous distribution of particles (Fig. 1D), and there were no 1,4-dioxane solvated CUR crystals in the form of hollow spherical crystals as had been reported (Wan et al., 2023) suggesting that the drug was successfully encapsulated in the carrier pores.

CUR showed strong characteristic crystal diffraction peaks at 5° to 30°, which was consistent with the previous report (Kan et al., 2024), and it was seen that CUR existed in the crystalline state; whereas, in CUR@γ-CD-MOFs, the diffraction peaks were similar to those of γ-CD-MOFs, and the characteristic peaks of the diffraction of CUR were basically disappeared (Fig. 1E), which indicated that CUR was distributed in the molecular or amorphous state in CUR@γ-CD-MOFs. In addition, CUR showed a heat-absorbing peak near 180 °C (Del Duca et al., 2024); the heat-absorbing peak near 180 °C still existed in the physical mixtures; whereas no heat-absorbing peaks appeared in both γ-CD-MOFs and CUR@γ-CD-MOFs at the similar positions (Fig. 1F), which indicated that the crystalline form of CUR in CUR@γ-CD-MOFs was altered, and the results are in agreement with the results of SEM and XRD tests.

The equilibrium solubility of CUR in CUR@γ-CD-MOFs was nearly 300-fold higher than the equilibrium solubility of CUR and nearly 20-fold higher than that of the CUR@γ-CD group (Fig. 1G), which indicated that the γ-CD-MOFs had a powerful solubilizing effect on CUR and the effect was significantly better than that of the γ-CD as a carrier alone, which was attributed to the extremely rich pore-like structure of the MOFs, thus bringing a large amount of CUR adsorbed in them in a solubilized fast molecular or amorphous state (Lv et al., 2024). In addition, we detected a pH value of 9.60 for an aqueous solution of 1 mg/mL of CUR@γ-CD-MOF, suggesting that γ-CD-MOF rapidly disintegrates in water and releases KOH, which undergoes a salt-forming reaction with CUR with phenolic hydroxyl groups to increase its solubility in water, and thus this property of γ-CD-MOF provides another way for it to improve the solubility of CUR.

### 3.2 β-CDNS has good encapsulation and solubilization properties for CUR

**Fig. 2:**
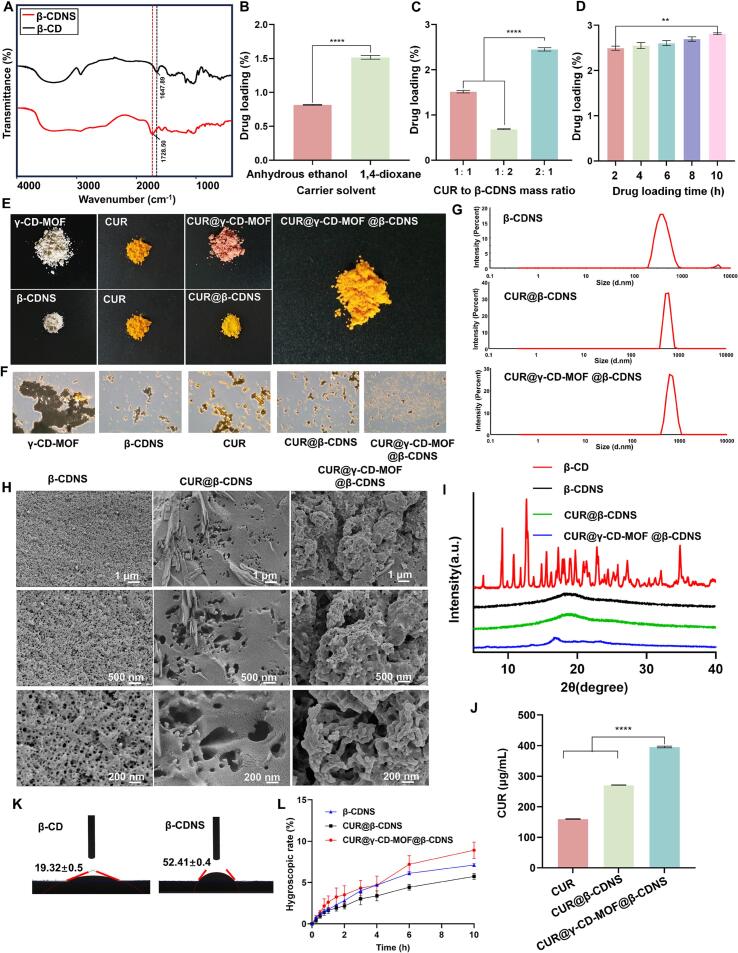
Preparation process optimization and characterization of CUR@γ-CD-MOF@β-CDNS. (A) Infrared spectra of β-CD and β-CDNS. (B) Effect of carrier solvent on drug loading of β-CDNS (****p < 0.0001; *n* = 3). (C) Effect of CUR to β-CDNS mass ratio on drug loading of β-CDNS (****p < 0.0001; n = 3). (D) Effect of drug loading time on drug loading of β-CDNS (**p < 0.01; n = 3). (E) The appearance of various powders under normal light. (F) Micrographs of various powders were magnified 200 times. (G) The particle size distribution profiles of the samples were measured using dynamic light scattering in INTENSITY mode. (H) Scanning electron micrographs at different magnifications. (I) Powder X-ray diffraction patterns. (J).Equilibrium solubility of CUR in water at 37 °C for each test group (****p < 0.0001; n = 3). (K) Contact angle test images. (L) Curves of hygroscopic rates with time for each test group (n = 3).

The use of crosslinking agents significantly affects the physicochemical properties of CDNS. CDNS prepared with cross-linking agents containing dianhydride structure such as homophthalic anhydride and EPI possessed water-absorbing and swelling properties, which could be transformed from the nanoparticle phase to the gel state for sustained and rapid drug release, whereas CDNS prepared with diphenyl carbonate did not possess water-absorbing and swelling properties (Dora et al., 2016; Gholibegloo et al., 2019). It has been reported that nanoparticle phase-transformation hydrogels can prevent skin and mucosal tissue damage and alleviate inflammation by forming a gel barrier in the mucosa, adsorbing inflammatory factors, and physically isolating the ulcerated surface of the local tissue (Chen et al., 2024a). Therefore, the preparation of CDNS-loaded CURs using EPI as a cross-linking agent is expected to solve the limitations of poor water solubility and chemical stability of CURs, and at the same time, combine with the carrier physically assisted therapeutic function, which provides a new strategy for the treatment of diseases of skin and mucosal tissues. The hydroxyl (OH) bending vibrational absorption peak at 1647 cm^−1^ disappeared after crosslinking of β-CD, and the ester carbonyl (C=O) stretching vibrational absorption peak at 1728 cm^−1^ appeared in the crosslinked β-CDNS (Fig. 2A), which suggests that β-CD reacts with the cross-linker via -OH and is -C=O for reticular linkage (Salem et al., 2023).

The preparation process of CUR@β-CDNS was examined and the drug loading with anhydrous ethanol as the carrier solvent was 0.82 ± 0.01 %, while the drug loading with 1,4-dioxane as the carrier solvent was 1.8 times higher than that of the anhydrous ethanol group (*p* < 0.0001) (Fig. 2B), which may be due to the better solubility of the drug in the 1,4-dioxane (Rasal et al., 2024). Continuing with the 1,4-dioxane as the carrier solvent, the drug loading increased with increasing drug mass ratio in the range of CUR to β-CDNS mass ratios from 1:2 to 2:1 (Fig. 2C), indicating that higher concentrations of the drug were more favorable for passive diffusion and were encapsulated in the molecular cavities of the cyclodextrins (Shawky et al., 2024; Zhang et al., 2024). Subsequently, the drug loading was carried out according to the mass ratio of CUR to β-CDNS of 2:1, and the loading time was from 2 to 10 h. The amount of drug loaded increased slightly with the prolongation of the loading time, and the amount of drug loaded after 10 h was significantly higher than that of the 2 h group (*p* < 0.01) (Fig. 2D). Based on the above analysis, the loading process was determined as 1,4-dioxane as the carrier solvent, the mass ratio of CUR to β-CDNS was 2:1, and the loading duration was 10 h.

Under normal light, β-CDNS and γ-CD-MOF appeared white, CUR appeared yellow, and CUR@β-CDNS appeared yellowish (Fig. 2E). While CUR@γ-CD-MOF appeared pink, which was due to the conjugation effect of the hydroxyl groups at both ends of the CUR molecule under alkaline conditions in which the electron cloud deviation occurs, so that CUR changed from yellow to red when the pH was greater than 8. However, the pH of the solution of CUR@γ-CD-MOF@β-CDNS (1 mg/mL) was 5.98, and the appearance color of its dried powder showed a yellow color that was almost indistinguishable from that of CUR powder, suggesting that the alkalized CUR in CUR@CD-MOF was reduced to its original molecular form due to the conversion of pH to acidity. In addition, because CUR@CD-MOF@CDNS is weakly acidic, it is suitable for dermal administration. Magnified 200×, γ-CD-MOF and β-CDNS appeared as fine powders, and CUR had a unique crystal structure, but CUR@β-CDNS did not show an obvious crystal structure, whereas CUR@γ-CD-MOF@β-CDNS had a combination of the structures of β-CDNS, γ-CD-MOF, and CUR@β-CDNS (Fig. 2F). In addition, no crystal structure of CUR was found in any of the drug-carrying nanocarriers, indicating that CUR was encapsulated into the carriers in an amorphous state.

The particle sizes of β-CDNS, CUR@β-CDNS, and CUR@γ-CD-MOF@β-CDNS were measured as 401.78 ± 12.38 nm, 536.06 ± 12.99 nm, and 744.53 ± 11.23 nm with PDI of 0.196 ± 0.033, 0.193 ± 0.05, and 0.204 ± 0.100, respectively, using dynamic light scattering (Fig. 2G). The PDI of all three samples was less than 0.3, showing that the particle size distribution was uniform and increased with more substances encapsulated in the nanosponges. The lyophilized β-CDNS had a three-dimensional porous structure, which was in line with the characteristics of nanosponges. Additionally, the percentages of C, H, N, and S in the currently prepared β-CDNS were 51.56 ± 0.16 %, 6.50 ± 0.08 %, 1.79 ± 0.04 %, and 0.76 ± 0.07 %, respectively, with the highest content of elemental C, less than 2 % of N, and less than 1 % of S. This suggests that the amount of residual triethanolamine and DMSO in the product is very small. However, in future processes it will be necessary to further remove the solvents used as completely as possible by repeated washing. After loading CUR into β-CDNS, the spatial structure became tight, but the porous structure was still seen. Co-loading of CUR and CUR@γ-CD-MOF into the nanosponges did not disrupt the porous structure of the nanosponges (Fig. 2H).

X-rays diffracted in β-CD crystals, showing multiple sharp wave peaks. In contrast, β-CDNS did not show sharp wave peaks and presented smooth wave peaks, suggesting that β-CDNS was successfully synthesized. The diffraction spectra of CUR@β-CDNS were similar to those of β-CDNS and did not have the characteristic diffraction peaks of CUR (Fig. 1E, Fig. 2I), which indicated that CUR was successfully encapsulated in the pores of β-CDNS in the non-crystalline state. Additionally, the characteristic peaks of γ-CD-MOF appeared in the CUR@γ-CD-MOF@β-CDNS group and the peak shape was similar to that of CUR@β-CDNS, but without the characteristic peaks of CUR, indicating that CUR@γ-CD-MOF maintained the structure of the carrier in β-CDNS but CUR was dispersed in an amorphous state in both γ-CD-MOF and β-CDNS.

The equilibrium solubility of CUR in PBS containing 1 % sodium dodecyl sulfate and 0.5 % Tween 80 was 159.85 ± 1.54 μg/mL, and the equilibrium solubility in CUR@β-CDNS and CUR@γ-CD-MOF@β-CDNS was 1.69 and 2.47 times higher than that of the free drug group, respectively, indicating that β-CDNS had a more obvious solubilizing effect on the CUR (Mognetti et al., 2012), while the incorporation of γ-CD-MOF significantly improved the drug in the solubility in the dispersion medium (*p* < 0.0001; Fig. 2J).

### 3.3 CUR@γ-CD-MOF@β-CDNS with weak gelation and bioadhesion properties

The cross-linking of β-CD to prepare β-CDNS increased the static contact angle (Fig. 2K), suggesting an increase in hydrophobicity, which could be attributed to the presence of cross-linking agent to reduce the polarity of β-CDNS, as well as the tight cross-linking between β-CDs, which makes it difficult for water molecules to enter between the molecular pores (Mele et al., 2011). Further hygroscopicity tests responded to this inference, and the hygroscopicity of β-CDNS, CUR@β-CDNS, and CUR@γ-CD-MOF@β-CDNS was less than 10 % within 10 h (Fig. 2L), indicating that all three formulations were not easy to absorb moisture and had relatively stable structures.

**Fig. 3:**
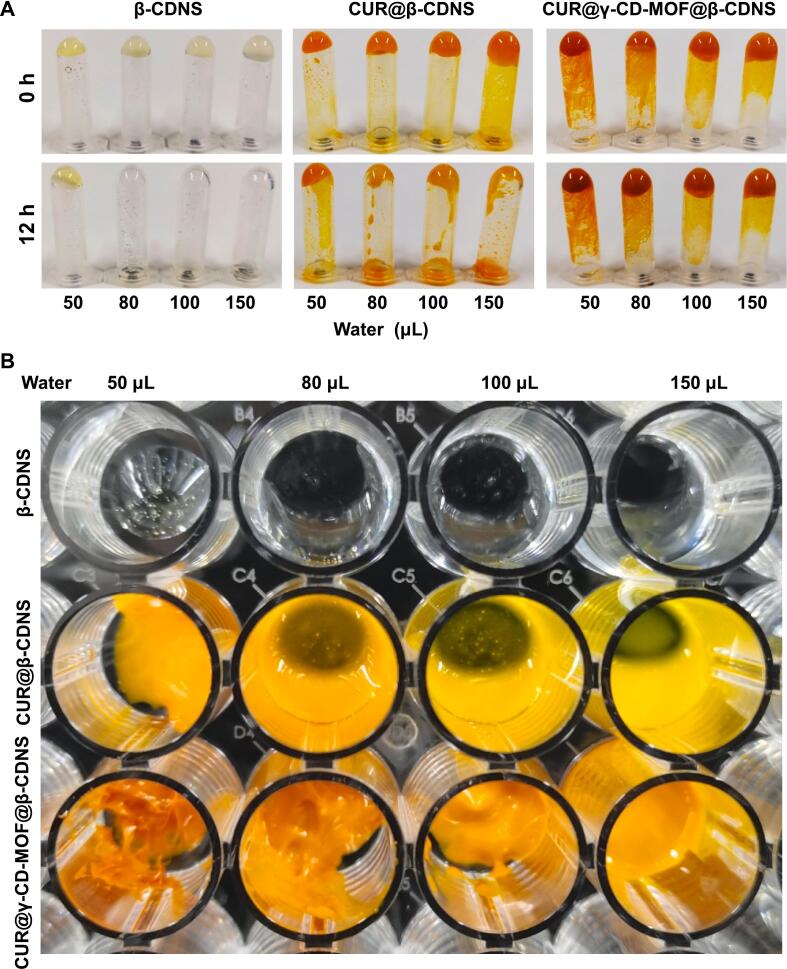
Hydrogel state of β-CDNS and its drug-carrying formulations. (A) Appearance of 100 mg of each test sample in a centrifuge tube mixed with different volumes of water to form a hydrogel and then inverted for 12 h. (B) Visualization of the hydrogels in the tubes placed for 12 h for each group.

β-CDNS and its drug-carrying formulations CUR@β-CDNS and CUR@γ-CD-MOF@β-CDNS were respectively dissolved in water to form hydrogels (El-Sayed et al., 2024). It was observed that the gel formed by the addition of 50 μL of pure water to the formulation of 100 mg was the strongest in adhesion, and did not fall off in the centrifuge tubes were inverted for 12 h, whereas all the groups with more water content showed gel slippage (Fig. 3), which further confirmed that the β-CDNS is weakly absorbent, but its gel state is adhesive and favorable for local administration.

**Fig. 4:**
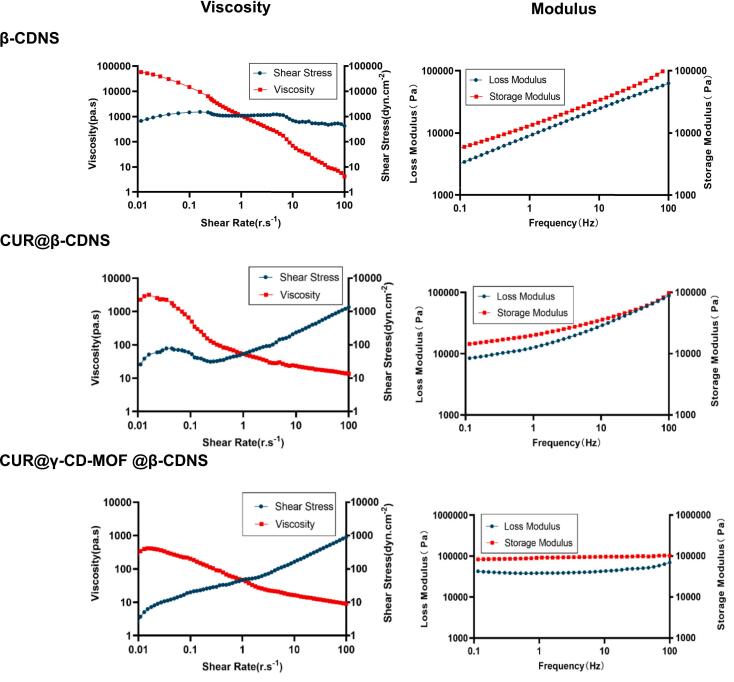
Rheological profiles of hydrogels formed with 100 mg of β-CDNS and its carrier formulations with 50 μL of water, respectively.

Gels were prepared by adding 50 μL of pure water to 100 mg of β-CDNS, CUR@β-CDNS, and CUR@γ-CD-MOF@β-CDNS, respectively, and were tested for rheology and viscoelasticity. The hydrogels formed by all three formulations showed a slow decrease in shear stress and a rapid decrease in shear viscosity with increasing shear rate, changing from the initial high-viscous material to a low-viscous material (Fig. 4), suggesting that the formulations had a shear-thinning behavior when in the gel state, presenting a non-Newtonian fluid characteristic, which is convenient to apply on the affected area for administration of the drug. In addition, the storage modulus was greater than the loss modulus for all three formulations. The storage modulus (G') characterizes the performance of the material in storing energy, which is related to the elasticity of the material, and the material with higher storage modulus can recover its original shape after deformation, showing solid-like properties; while the loss modulus (G") characterizes the performance of the material in dissipating energy, which is related to the viscosity of the material, and the material with higher loss modulus can dissipate the energy in the form of heat when subjected to deformation, showing liquid-like properties. The results indicate that the formulations tend to be solid in nature, have excellent physical stability and adhere to the localized site of administration without the concern of flow affecting the effectiveness of drug delivery.

**Fig. 5:**
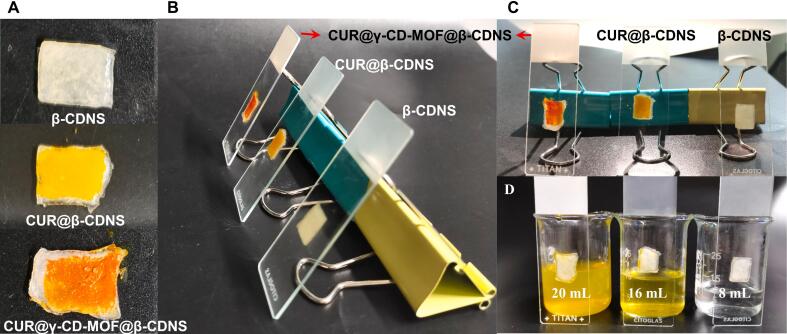
Bioadhesion of hydrogels of β-CDNS and its carrier formulations. (A) β-CDNS and its carrier formulations respectively coated on the stratum corneum of isolated mouse skins. (B) Isolated mouse skins containing β-CDNS and its carrier formulations were respectively placed at an inclined position on a slide with the dermal side adhered to them. (C) Volume of water required to completely rinse off the hydrogel on the stratum corneum of the skin of each group.

The results of subsequent skin adhesion tests confirmed the superiority of β-CDNS as a carrier for topical drug delivery. β-CDNS, CUR@β-CDNS, and CUR@γ-CD-MOF@β-CDNS hydrogels remained tightly adherent to isolated mouse skin after being left at an inclined 45° angle for 10 min, whereas the gels were washed out with a syringe at a rate of 10 mL/min. In the same rinsing of the gel on the skin, the β-CDNS, CUR@β-CDNS, and CUR@γ-CD-MOF@β-CDNS gels took 8, 16, and 20 mL of water, respectively, to be completely rinsed off, showing that the CUR@γ-CD-MOF@β-CDNS hydrogel has the strongest skin adhesion, which may be attributed to the stronger hygroscopicity brought by γ-CD-MOFs (Fig. 5), as well as the strong adsorption properties due to its porous structure (Li et al., 2023).

### 3.4 CUR@γ-CD-MOF@β-CDNS significantly improved the in vitro release and percutaneous permeation of CUR

**Fig. 6:**
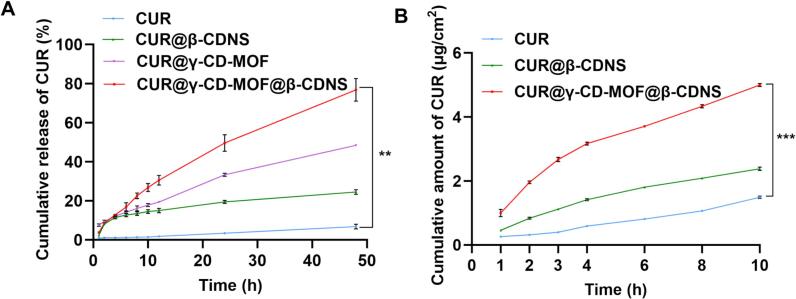
In vitro cumulative release (A) and transdermal permeation (B) profiles of free CUR (CUR) and its carrier formulation groups. **p < 0.01, ***p < 0.001. *n* = 3.

Loading CUR into β-CDNS improved its in vitro release. Within 4 h, the drug release of CUR@β-CDNS and CUR@γ-CD-MOF@β-CDNS groups mainly originated from β-CDNS, so the drug release was not much different, but both were significantly larger than that of the free CUR group, which was attributed to the improved solubility of CUR by β-CDNS. However, since the drug loading of β-CDNS was lower than that of γ-CD-MOF, in the CUR@β-CDNS and CUR@γ-CD-MOF@β-CDNS groups containing the same mass of drug, the mass of β-CDNS used in the former group was larger while the drug concentration was smaller, resulting in the drug not rivaling the latter group in terms of the diffusion rate. In contrast, in the CUR@γ-CD-MOF@β-CDNS group, CUR was rapidly released with the continuous disintegration of γ-CD-MOF, and the release rate of CUR was significantly greater than that of the CUR@β-CDNS group and amounted to 76.73 ± 5.76 % at 48 h (Fig. 6A), which was 11.25 times higher than that of the free drug group and 3.13 times higher than that of the CUR@β-CDNS group, respectively. The above results confirmed that the combination of γ-CD-MOF and β-CDNS as a delivery vehicle for CUR significantly improved the drug release, thus providing a strong guarantee for further increased in vivo absorption (Buschmann and Schollmeyer, 2002; Swaminathan et al., 2007).

The in vitro transdermal permeation performance of each test group responded to the results of the in vitro release test. The free drug group had the lowest in vitro transdermal permeation efficiency due to the poor water solubility of CUR. The high drug loading and rapid drug release of CUR@γ-CD-MOF@β-CDNS, which formed a higher drug concentration gradient on the skin surface, produced a stronger permeation enhancement effect and obtained higher cumulative transdermal permeation of the drug than that of the CUR@β-CDNS group (p < 0.001) (Fig. 6B).

**Fig. 7:**
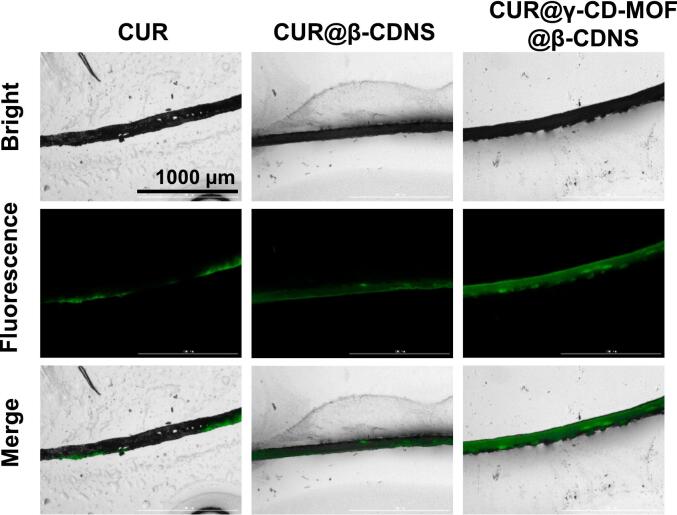
Fluorescence micrographs of frozen sections of isolated mouse skins respectively subjected to 10 h of in vitro transdermal permeation with free CUR (CUR) and its drug-carrying formulations.

With the property that CUR has spontaneous green fluorescence, we further analyzed the distribution of the drug in the skin. After 10 h of transdermal permeation, the CUR@γ-CD-MOF@β-CDNS group showed the strongest fluorescence, followed by the CUR@β-CDNS group, and the free CUR group showed the weakest fluorescence in the skin tissues (Fig. 7), which was consistent with the trend of the amount of in vitro transdermal permeation of the drug, suggesting that the permeation-enhancing effect of the carriers increased the distribution of the drug in the skin tissues, which was thus able to enhance the local therapeutic effect of the drug. In addition, the fluorescence of the CUR@β-CDNS group and the CUR@γ-CD-MOF@β-CDNS group was more evenly distributed in the skin tissue than that of the free drug group, which was attributed to the excellent bioadhesive property of the β-CDNS hydrogel, which achieved a continuous and uniform permeation of the drug into the local tissues by stabilizing the drug at the site of drug administration and maintained the effective drug concentration in the local tissues, which helped to avoid poor efficacy or toxic side effects that may be caused by uneven absorption of the drug.

## 4 Conclusion

Using γ-CD-MOF and β-CDNS together as a composite carrier of CUR significantly increased the drug loading capacity of the insoluble drug CUR and improved its water solubility, thus facilitating the local absorption of the drug. CUR@γ-CD-MOF@β-CDNS absorbs water to form a weak gel with good bioadhesion after being applied to wounds or sores with high exudate, and possesses three therapeutic effects by absorbing exudate, releasing drugs and forming a gel layer with physical protection. In addition, the composite carrier is less hygroscopic in nature, which can reduce the counter-absorption of water by the sores, thus it is more suitable for the topical treatment of the affected areas or wounds with more exudate than ordinary hydrogels and water-based ointments, and the composite carrier is free of other additives, which predicts that it has excellent biocompatibility. In short, this composite carrier integrating high drug loading, increased drug release, permeation enhancement, bioadhesion, and fewer additives provides a worthwhile option for improving the efficacy of topical administration of insoluble drugs.

## CRediT authorship contribution statement

**Songting Li:** Validation, Supervision, Resources, Project administration, Funding acquisition, Conceptualization. **Meng Long:** Project administration, Writing – original draft. **Jiaqi Li:** Software, Investigation, Data curation. **Yongtai Zhang:** Validation, Methodology, Conceptualization, Writing – review & editing. **Nianping Feng:** Supervision, Resources, Investigation, Conceptualization. **Zhicheng Zhang:** Validation, Supervision, Resources, Methodology, Investigation, Funding acquisition, Writing – review & editing.

## Declaration of competing interest

The authors declare that they have no known competing financial interests or personal relationships that could have appeared to influence the work reported in this paper.

## Data Availability

Data will be made available on request.

## References

[bb0005] Ansari K.A., Vavia P.R., Trotta F. (2011). Cyclodextrin-based nanosponges for delivery of resveratrol: in vitro characterisation, stability, cytotoxicity and permeation study. AAPS PharmSciTech.

[bb0010] Buschmann H.J., Schollmeyer E. (2002). Applications of cyclodextrins in cosmetic products: a review. J. Cosmet. Sci..

[bb0015] Chen J., An X., Xu L. (2024). Adhesive nanoparticle-in-microgel system with ROS scavenging capability and hemostatic activity for postoperative adhesion prevention. Small.

[bb0020] Chen L.H., Chen T., Zhao R.N. (2024). Physical properties and antioxidant activity of curcumin-zinc metal-organic frameworks. Food Chem..

[bb0025] Del Duca G., Parisi E., Artusio F. (2024). A crystal engineering approach for rational design of curcumin crystals for Pickering stabilization of emulsions. Food Res. Int..

[bb0030] Dora C.P., Trotta F., Kushwah V. (2016). Potential of erlotinib cyclodextrin nanosponge complex to enhance solubility, dissolution rate, in vitro cytotoxicity and oral bioavailability. Carbohydr. Polym..

[bb0035] El-Sayed S.E., Abdelaziz N.A., El-Housseiny G.S. (2024). Nanosponge hydrogel of octadecyl 3-(3,5-di-tert-butyl-4-hydroxyphenyl) propanoate of Alcaligenes faecalis. Appl. Microbiol. Biotechnol..

[bb0040] Forte J., Gioia Fabiano M., Grazia Ammendolia M. (2024). Bioactive pH-sensitive nanoemulsion in melanoma cell lines. Int. J. Pharm..

[bb0045] Garg A., Lai W.C., Chopra H. (2024). Nanosponge: a promising and intriguing strategy in medical and pharmaceutical. Sci. Heliyon.

[bb0050] Gholibegloo E., Mortezazadeh T., Salehian F. (2019). Improved curcumin loading, release, solubility and toxicity by tuning the molar ratio of cross-linker to β-cyclodextrin. Carbohydr. Polym..

[bb0055] Ghosh M., Sarkar N. (2024).

[bb0060] Gigliotti C.L., Ferrara B., Occhipinti S. (2017). Enhanced cytotoxic effect of camptothecin nanosponges in anaplastic thyroid cancer cells in vitro and in vivo on orthotopic xenograft tumors. Drug Deliv..

[bb0065] Hajimirzaei P., Eyni H., Razmgir M. (2024). The analgesic effect of curcumin and nano-curcumin in clinical and preclinical studies: a systematic review and meta-analysis Naunyn Schmiedebergs. Arch. Pharmacol..

[bb0070] He S., Wu L., Li X. (2021). Metal-organic frameworks for advanced drug delivery. Acta Pharm. Sin. B.

[bb0075] Hefayathullah M., Singh S., Ganesan V. (2024). Metal-organic frameworks for biomedical applications: a review. Adv. Colloid Interf. Sci..

[bb0080] Kan G., Zi Y., Shi C. (2024). Interaction of curcumin with four types of gelatins in nanoparticles: Mechanism and application for emulsion stabilization. Food Hydrocoll..

[bb0085] Kandaswamy K., Prasad Panda S., Subramanian R. (2024). Synergistic berberine chloride and Curcumin-Loaded nanofiber therapies against Methicillin-Resistant Staphylococcus aureus Infection: Augmented immune and inflammatory responses in zebrafish wound healing. Int. Immunopharmacol..

[bb0090] Kru Kle-Be Rziṇa K.N., Lends A., Boguszewska-Czubara A. (2024). Cyclodextrin metal-organic frameworks as a drug delivery system for selected active pharmaceutical ingredients. ACS Omega.

[bb0095] Li W., Gan Y., Li Y. (2023). Enhancing propellant performance through intermolecular interactions: cyclodextrin-based MOF loading in nitrocellulose. Phys. Chem. Chem. Phys..

[bb0100] Liu Y., Yin R., Tian Y. (2024). Curcumin nanopreparations: recent advance in preparation and application. Biomed. Mater..

[bb0105] Liu C., Tian C., Guo J. (2024). Research Progress of Metal-Organic Frameworks as Drug delivery Systems. ACS Appl. Mater. Interfaces.

[bb0110] Lu B., Zhong Y., Zhang J. (2024). Curcumin-based ionic liquid hydrogel for topical transdermal delivery of curcumin to improve its therapeutic effect on the psoriasis mouse model. ACS Appl. Mater. Interfaces.

[bb0115] Lv F., Fang H., Huang L. (2024). Curcumin equipped nanozyme-like metal-organic framework platform for the targeted atherosclerosis treatment with lipid regulation and enhanced magnetic resonance imaging capability. Adv. Sci. (Weinh.).

[bb0120] Mele A., Castiglione F., Malpezzi L. (2011). HR MAS NMR, powder XRD and Raman spectroscopy study of inclusion phenomena in βCD nanosponges. J. Incl. Phenom. Macrocycl. Chem..

[bb0125] Mognetti B., Barberis A., Marino S. (2012). In vitro enhancement of anticancer activity of paclitaxel by a Cremophor free cyclodextrin-based nanosponge formulation. J. Incl. Phenom. Macrocycl. Chem..

[bb0130] Nagargoje A.A., Deshmukh T.R., Shaikh M.H. (2024). Anticancer perspectives of monocarbonyl analogs of curcumin: a decade (2014-2024) review. Arch. Pharm. (Weinheim).

[bb0135] Nunes Y.C., Mendes N.M., Pereira de Lima E. (2024). Curcumin: a Golden Approach to healthy Aging: a Systematic Review of the evidence. Nutrients.

[bb0140] Poornima G., Deepa M., Devadharshini M. (2024). In-situ synthesis and evaluation of anti-bacterial efficacy and angiogenesis of curcumin encapsulated lipogel dermal patch for wound healing applications. Biomater. Adv..

[bb0145] Prasain J.K., Barnes S., Attaur R. (2016). Studies in Natural Products Chemistry.

[bb0150] Pyrak B., Rogacka-Pyrak K., Gubica T. (2024). Exploring cyclodextrin-based nanosponges as drug delivery systems: understanding the physicochemical factors influencing drug loading and release kinetics. Int. J. Mol. Sci..

[bb0155] Qin Z., Jiang Q., Zou Y. (2024). Synthesis of nanosized γ-cyclodextrin metal-organic frameworks as carriers of limonene for fresh-cut fruit preservation based on polycaprolactone nanofibers. Small.

[bb0160] Rasal R.K., Badsha I., Shellaiah M. (2024). Fabrication of curcumin-based electrochemical nanosensors for the detection of environmental pollutants: 1,4-dioxane and hydrazine. Biosensors (Basel).

[bb0165] Ruan S., Li J., Ruan H. (2024). Microneedle-mediated nose-to-brain drug delivery for improved Alzheimer’s disease treatment. J. Control. Release.

[bb0170] Ruan H., Long M., Li J. (2024). Sustained-release hydrogen-powered bilateral microneedles integrating CD-MOFs for in situ treating allergic rhinitis. Adv. Healthc. Mater..

[bb0175] Salem Y.Y., Hoti G., Sammour R.M.F. (2023). Preparation and evaluation of βcyclodextrin-based nanosponges loaded with Budesonide for pulmonary delivery. Int. J. Pharm..

[bb0180] Sghier K., Mur M., Veiga F. (2024). Novel therapeutic hybrid systems using hydrogels and nanotechnology: a focus on nanoemulgels for the treatment of skin diseases. Gels.

[bb0185] Shawky H., Fayed D.B., Ibrahim N.E. (2024). pH-tailored delivery of a multitarget anticancer benzimidazole derivative using a PEGylated β-cyclodextrin-curcumin functionalized nanocomplex. Biomater. Adv..

[bb0190] Shende P., Kulkarni Y.A., Gaud R.S. (2015). Acute and repeated dose toxicity studies of different β-cyclodextrin-based nanosponge formulations. J. Pharm. Sci..

[bb0195] Sheng Y, Yu Q, Huang Y et al. (2023) Pickering Emulsions Enhance Oral Bioavailability of Curcumin Nanocrystals: the effect of Oil Types Pharmaceutics 15 doi:doi:10.3390/pharmaceutics15051341. PMC10221895 37242583

[bb0200] Singh P., Mahar R. (2024). Cyclodextrin in drug delivery: Exploring scaffolds, properties, and cutting-edge applications. Int. J. Pharm..

[bb0205] Swaminathan S., Vavia P.R., Trotta F. (2007). Formulation of betacyclodextrin based nanosponges of itraconazole. J. Incl. Phenom. Macrocycl. Chem..

[bb0210] Wan X., Chen Y., Cao Y. (2023). Reversible intramolecular proton transfer in curcumin crystals and nonlinear size correlation. Cryst. Growth Des..

[bb0215] Wu T., Hou X., Li J. (2021). Microneedle-mediated biomimetic cyclodextrin metal organic frameworks for active targeting and treatment of hypertrophic scars. ACS Nano.

[bb0220] Wu X.X., Law S.K., Ma H. (2024). Bio-active metabolites from Chinese Medicinal Herbs for treatment of skin diseases. Nat. Prod. Res..

[bb0225] Yusuf H., Savitri O.M.N., Al-Khalifi N.N. (2024). Cellulose- and saccharide-based orally dispersible thin films transform the solid states and dissolution characteristics of poorly soluble curcumin. Adv. Pharmacol. Pharm. Sci..

[bb0230] Zhang Z., Miao W., Ji H. (2024). Interaction of zein/HP-β-CD nanoparticles with digestive enzymes: Enhancing curcumin bioavailability. Food Chem..

[bb0235] Zhao Q., Gu N., Li Y. (2024). Self-assembled gel microneedle formed by MS deep eutectic solvent as a transdermal delivery system for hyperpigmentation treatment. Mater. Today Bio..

